# Does the Naked Neck Meat Type Chicken Yield Lower Methionine Requirement Data?

**DOI:** 10.3390/ani5020151

**Published:** 2015-03-25

**Authors:** Daulat R. Khan, Christian Wecke, Frank Liebert

**Affiliations:** Division Animal Nutrition Physiology, Department of Animal Sciences, Georg-August-University, Kellnerweg 6, 37077 Goettingen, Germany; E-Mails: dkhan@gwdg.de (D.R.K.); cwecke@gwdg.de (C.W.)

**Keywords:** naked neck meat type chicken, N balance studies, amino acid efficiency, methionine requirement

## Abstract

**Simple Summary:**

It was hypothesized that naked neck chickens could have a lower methionine requirement according to their reduced feather coverage. The hypothesis was examined by nitrogen balance studies and non-linear model application for estimating methionine requirement data of naked neck chickens. It was concluded that naked neck birds do not require less methionine than normally-feathered birds.

**Abstract:**

Methionine (Met) requirement studies with homozygous (*Na/Na*) and heterozygous (*Na/na*) naked neck meat type chicken utilized 144 birds of average weight (50% each genotype and sex) within two N balance experiments involving both the starter (d10–20) and grower period (d25–35). The birds were randomly allotted to five experimental diets with graded protein supply and Met as the limiting amino acid. The proportion of native feed protein sources (soy protein concentrate, maize, wheat, fishmeal and wheat gluten) was kept constant to ensure a uniform protein quality in all diets. The Met requirement depending on genotype, sex, age period and growth performance (protein deposition) was estimated using a non-linear modeling procedure of N utilization in monogastric animals. On average, 0.47% (*Na/Na*) and 0.45% (*Na/na*) dietary Met was established as adequate in the starter diet, as well as 0.37% (*Na/Na*) and 0.36% (*Na/na*) Met in the grower diet for both of the sexes. In conclusion, the Met requirement of the naked neck chicken is not significantly different from its normally-feathered counterparts. In addition, the low feather production was not reflected by reduced requirement for Met in naked neck birds. However, these conclusions are valid only at the given Met:Cys ratio (1:1) in the experimental diets.

## 1. Introduction

Previous protein requirement studies in naked neck birds came to inconsistent results. Ajang *et al.* [1] suggested that the degree of feathering in fast and slow feathering broilers may influence the crude protein (CP) requirement of chickens. Yalcin *et al.* [2] observed that naked neck birds did not require less dietary protein because of their reduced feather covering. Nir [3] also suspected that the concentration of sulfur containing amino acid (SAA) in feather protein is about double than in body protein, and that the variation in the feathering may have a major effect on optimal dietary amino acid (AA) composition. Pesti *et al.* [4] examined this hypothesis by feeding conventional homozygous (*na/na*) and heterozygous (*Na/na*) birds between 38 and 42 d of age with diets ranging between 5.4 and 7 g SAA/kg. It was concluded that the growth rate of both genotypes was similarly influenced by the dietary supply of SAA under uniform climate conditions.

The present studies aimed at determining the Met requirement data for both homozygous (*Na/Na*) and heterozygous (*Na/na*) naked neck meat type chicken depending on age and gender by application of a non-linear N utilization model [5,6,7,8,9]. This focus on Met takes into account that feather protein contains more than 10 times the quantity of Cys. However, the metabolic need for Cys is completely satisfied by degradation of Met, if the AA is sufficiently available from feed.

## 2. Materials and Methods

### 2.1. Stock and Husbandry

The experiments were conducted at the Division of Animal Nutrition Physiology, Department of Animal Sciences at Georg-August-University of Goettingen with the approval of the Animal Welfare Law Committee of Lower Saxony, Germany.

The birds were reared under standardized housing and feeding regimes up to the start of the experiments. Afterwards, a total of 144 birds of average weight (72 *Na/Na* and 72 *Na/na*, each 50% male and female) were selected for the N balance experiments involving both starter (d10-20) and grower period (d25-35). The birds were individually housed in metabolic cages with wire floor, equipped with individual feeder and self-drinking system. The temperature in the experimental room was gradually reduced from 32 to 23 °C with increasing age. Humidity was maintained between 60%–70% and monochromatic light was provided for 23 h following 1 h darkness.

### 2.2. Diets, Sampling and Analyses

Chickens of both genotypes and sexes were randomly allotted to five pelleted experimental diets between 10% and 38% crude protein (CP) created by application of principles of the diet dilution technique [10]. Details about the composition and nutritional value of the diets have already been reported by Khan *et al.* [11]. However, it is essential to highlight that all diets both in the starter and grower period had a constant mixture of the utilized native feed protein sources soy protein concentrate (SPC), maize, wheat, fishmeal and wheat gluten (Table 1). Consequently, a consistent dietary protein quality was ensured and lastly confirmed by the results obtained, following feeding of all five CP graded diets to animals of both genotypes and sexes and all age periods, respectively. According to the needs of optimal statistical power for estimation of model parameters [11], 6 to 10 replications per diet under study were generated.

**Table 1:** Constant mixture of feed protein sources in the experimental diets with graded dietary protein supply [11].

Ingredients	Soy Protein Concentrate	Maize	Wheat	Fish Meal	Wheat Gluten
Percent of feed protein mixture	39.8	25.9	19.9	7.5	6.9

Consequently, the dietary AA pattern was kept unchanged independent on protein level in the diets of both age periods. To create optimal dietary AA ratios based on conventional feed ingredients only, a feed optimization program (Fumi for Windows 4, HYBRIMIN^®^ Computer + Programme GmbH & Co. KG, Hessisch Oldendorf, Germany) was applied. The dietary AA ratios were adjusted to be near to the ideal AA ratio (IAAR) as derived from literature data [8], except for Met. Met supply was set as limiting AA (LAA) in the diets (Table 2) to justify further modeling of Met requirement data.

**Table 2:** Amino acid (AA) composition of the experimental diets and recommended ideal amino acid ratio (IAAR) in diets for growing chicken.

	Dietary AA Concentration		Ideal Dietary Ratios for Individual Amino Acids Related to Lys (Lys = 100)		
	g/16 g N	Ratio Relative to Lys	Mean of Literature Data [8]	GRSS [13]	NRC [14]
Lysine (Lys)	5.09	100	100	100	100
Methionine (Met)	1.44	28	40	37	42
Methionine + Cysteine (Met + Cys)	2.91	57	74	71	72
Threonine (Thr)	3.63	71	66	67	74
Tryptophan (Trp)	0.96	19	16	16	18
Arginine (Arg)	6.26	123	105	108	110
Histidine (His)	2.36	46	34	32	32
Isoleucine (Ile)	4.03	79	69	69	73
Valine (Val)	4.24	83	80	-	82
Leucine (Leu)	7.50	147	110	112	109
Phenylalanine (Phe)	4.71	93	66	65	65
Phenylalanine + Tyrosine (Phe + Tyr)	8.35	164	120	118	122

Two N balance experiments during starter and grower period were conducted for each genotype and sex, respectively. The individual experimental period was divided into an adaptation period (5 d) and 2 consecutive collecting periods (each 5 d). Excreta collection was conducted 2 times a day to prevent ammonia losses from un-acidified excreta. Excreta samples were immediately frozen and stored at −20 °C until further analysis. Dietary ingredients, experimental diets and excreta were analyzed according to the German standards [12]. The N content was quantified due to the Dumas method (Leco^®^ LP-2000, Leco^®^ Instrument GmbH, Kirchheim, Germany) and CP was calculated with factor 6.25. AA analyses were conducted by ion-exchange chromatography (Biochrom^®^ 30, Biochrom Ltd. Cambridge, England) following acid hydrolysis with and without an oxidation step for quantitative determination of sulfur-containing amino acids.

According to current applications of our modeling procedure based on individual AA efficiency [5,7,9], Met requirement data for given NR were derived as follows:

(1) LAAI = [lnNR_max_T − ln (NR_max_T − NR)]/16·bc^−1^

where LAAI = daily intake of limiting amino acid (mg/BW_kg_^0.67^); NR_max_T = theoretical daily maximum for NR (mg/BW_kg_^0.67^); NR = nitrogen retention (mg/BW_kg_^0.67^); b = slope of the N retention curve (indicating the feed protein quality independent on N intake); c = concentration of the LAA in the dietary protein (g/16 g N); bc^−1^ = slope between c and b (model parameter, indicating the dietary LAA efficiency). The multiplier 16 results from LAA concentration in the dietary protein (g/16 g N).

Further modeling of Met requirement data according to Equation (1) utilized both age, genotype and sex specific model parameters (Table 3) and corresponding average of protein quality parameter b as reported by Khan *et al.* [11]. Due to real growth performance data, the desired daily body protein deposition (PD = ND·6.25; Table 4 and Table 5) was adapted to approximately 60%, 70% and 80% of the theoretical maximum (ND_max_T). During starter period, 500 g mean BW was applied for modeling both in males and in females, respectively. According to lower growth potential during grower period, for female chickens 1400 g mean BW was applied (Males 1500 g).

**Table 3:** Model parameters as derived from N balance experiments with fast growing naked neck chicken depending on age period, genotype and sex [11].

	Starter Period (d10-20)				Grower Period (d25-35)			
	*Na/Na*		*Na/na*		*Na/Na*		*Na/na*	
	Males	Females	Males	Females	Males	Females	Males	Females
NMR	262	348	224	392	341	384	346	395
NR_max_T	3763	3857	3965	4049	3397	2881	3512	3034
ND_max_T	3501	3509	3741	3657	3056	2497	3166	2639
b	288	267	274	248	291	356	282	329
bc^−1^	200	185	190	172	202	247	196	228

NMR = daily N maintenance requirement (mg/BW_kg_^0.67^); NR_max_T = theoretical maximum of daily N retention (mg/BW_kg_^0.67^); ND_max_T = theoretical maximum of daily N deposition (mg/BW_kg_^0.67^); b = model parameter assessing the dietary protein quality (b∙10^6^); bc^−1^ = model parameter assessing the dietary efficiency of Met as LAA (bc^−1^∙10^6^).

## 3. Results and Discussion

### Modeling of Met Requirement

Results of modeling Met requirement data for graded daily PD and feed intake within starter and grower period are summarized in Table 4 and Table 5.

**Table 4:** Modeling of Met requirement data for male naked neck (*Na/Na*; *Na/na*) meat type chicken in starter and grower periods, depending on daily body protein deposition (PD) and predicted daily feed intake (Mean BW: 500 g for starter period; 1500 g for grower period).

Item		*Na/Na*			*Na/na*			*Na/Na*			*Na/na*		
		Starter						Grower					
PD (g/d)		8	10	12	8	10	12	15	17.5	20	15	17.5	20
Met efficiency (bc^−1^)		200	200	200	190	190	190	202	202	202	196	196	196
Met requirement (mg/BW_kg_^0.67^/d)		295	428	666	277	394	576	315	403	527	308	391	502
Met content needed in the diet (%) depending on feed intake													
Feed intake (g/d)													
Starter	Grower												
50	120	0.37	0.54	0.84	0.35	0.50	0.72	0.34	0.44	0.58	0.34	0.43	0.55
60	130	0.31	0.45	0.70	0.29	0.41	0.60	0.32	0.41	0.53	0.31	0.39	0.51
70	140	0.26	0.38	0.60	0.25	0.35	0.52	0.30	0.38	0.49	0.29	0.37	0.47
80	150	0.23	0.34	0.52	0.22	0.31	0.45	0.28	0.35	0.46	0.27	0.34	0.44

**Table 5:** Modeling of Met requirement data for female naked neck (*Na/Na*; *Na/na*) meat type chicken in starter and grower periods, depending on daily body protein deposition (PD) and predicted daily feed intake (Mean BW: 500 g for starter period; 1400 g for grower period).

Item		*Na/Na*			*Na/na*			*Na/Na*			*Na/na*		
		Starter						Grower					
PD (g/d)		8	10	12	8	10	12	12	14	16	12	14	16
Met efficiency (bc^−1^)		185	185	185	172	172	172	247	247	247	228	228	228
Met requirement (mg/BW_kg_^0.67^/d)		324	468	721	332	469	691	277	354	467	276	348	445
Met content needed in the diet (%) depending on feed intake													
Feed intake (g/d)													
Starter	Grower												
50	120	0.41	0.59	0.91	0.42	0.59	0.87	0.29	0.37	0.49	0.29	0.36	0.47
60	130	0.34	0.49	0.75	0.35	0.49	0.72	0.27	0.34	0.45	0.27	0.33	0.43
70	140	0.29	0.42	0.65	0.30	0.42	0.62	0.25	0.32	0.42	0.25	0.31	0.40
80	150	0.25	0.37	0.57	0.26	0.36	0.54	0.23	0.30	0.39	0.23	0.29	0.37

During the starter period, 0.45% Met for *Na/Na* and 0.41% Met in the diet for *Na/na* chicken was observed as requirement level for male chicken, assuming 10 g daily body PD and 60 g daily feed intake (Table 4). In the grower period, 0.41% Met (*Na/Na*) and 0.39% Met (*Na/na*) in the diet were needed at 130 g daily feed intake to yield 17.5 g daily body PD. However, the results of modeling Met requirements need some further discussion in general, due to observed effects of varying model parameters as applied (Table 3). In contrast to expectation, higher Met efficiency data (bc^−1^) in male Na/Na chicken (Table 4) did not provide lower Met requirement data for an equal level of PD. This observation seems to be misleading, but originated from application of individual estimates of NMR and NR_max_T (Table 3) depending on genotype, sex and age period, respectively. Similar observations are shown for female chicken (Table 5). Consequently, under circumstances as described the calculation of Met efficiency is also under the influence of variation of model parameters NMR and NR_max_T, respectively. It can be discussed to eliminate this factor of influence by application of averaged “working values” for these model parameters. However, before applying such averaged values it is a precondition to create an approach for statistical evaluation of observed differences between estimates for NMR and NR_max_T, respectively. Ongoing bio-statistical research in our Division is dealing with this matter for future application.

**Figure 1:**
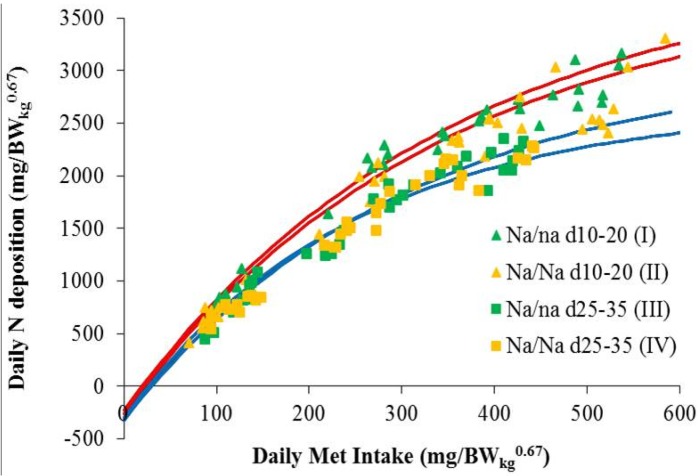
Daily N deposition (ND), depending on Met intake (MetI) and age period of male naked neck chickens. NR_max_T = theoretical maximum for daily N retention (mg/BW_kg_^0.67^); e = basic number of natural logarithm; bc^−1^ = efficiency of Met utilization; NMR = daily N maintenance requirement (mg/BW_kg_^0.67^).

Figure 1 summarizes the observed Met response curves for male chickens of both genotypes and age periods under study.

Generally, comparisons between our results of modeling and current recommendations are questionable according to the direct effect both of modulated PD and of Met efficiency data on our estimates. However, for male chicken at 10 g/d PD and 60 g/d feed intake our data are in the range of GRRS [13] recommendations (0.44%). For female naked neck meat type chickens during starter period, modeling data are slightly higher than German recommendations. The NRC [14] recommendations (0.50% and 0.52%) are higher than our data or corresponded to raised PD. In addition, current observations for the starter period are also lower than concluded by Liebert *et al.* [15] for fully feathered chicken (0.52%). For grower chickens with aimed PD of 17.5 g/d (male) and 14 g/d (female), which correspond to approximately 70% of the theoretical maximum (ND_max_T), the derived Met requirement data for male naked neck chickens are in line with GRRS [13] and NRC [14] recommendations (0.39% and 0.38%). But it was lower than recommended by Dirain and Waldroup [16] and Kalinowski *et al.* [17] for 3–6 week old chickens (0.44% and 0.46%). According to the lower PD potential of female chickens, the obtained Met requirement estimates generally declined.

It is well documented that derived AA requirements of growing chickens depend on many factors such as genotype, gender, age and investigated response parameters [18,19,20,21,22]. The applied modeling approach involves both the variation of daily protein deposition and predicted feed intake parameters. Taking into account these factors of influence yields a remarkable modulation of the optimal Met concentration in the diet (Table 4 and Table 5). This fact may create difficulties when the results of modeling are compared with recommendations, which do not take these important factors into account. Otherwise, this opportunity is a significant advantage of the modeling approach presented.

For comparison of the current data with results in naked neck genotypes, the available database is extremely scarce. However, Yalçin *et al.* [2] concluded that the needed Met content in diets for *Na/na* birds is not different from full-feathered chickens. Furthermore, Yalcin *et al.* [23] conducted experiments in heterozygous naked neck (*Na/na*) chicken to study the response to different dietary Met concentrations from 0.57% and 0.33% under spring and summer conditions, respectively. They concluded that the Met content needed in diets of *Na/na* birds did not differ from that of their normally-feathered counterparts under both ambient temperature conditions. In addition, no interaction between genotype and dietary Met concentration was observed. These observations are in agreement with our current results. Indifferent requirement data for Met by naked neck birds compared to normally-feathered counterparts are also supported by investigations of Pesti *et al.* [24].

Additionally, Yalçin *et al.* [2] observed no significant effect on carcass yield, but witnessed a significant effect of the 2nd order polynomial coefficient of dietary Met over breast muscle yield. Furthermore, higher abdominal fat mass in birds receiving a low Met diet was observed. Moreover, Deeb and Cahaner [25] stated a 4% and 8% higher breast meat percentage for both naked neck genotypes in *Na/na* and *Na/Na* over their normally-feathered sibs. They also found that the *Na/na* and *Na/Na* gained 4.5% and 8.1% more BW in grower period than *na/na* birds. Huyghebaert and Pack [26] identified higher slaughter yields and breast meat yields in naked neck chicken compared to normally-feathered birds and reduced fat deposition due to sulfur containing amino acid addition from 2–5 weeks of age. Regarding utilization of the spared Met, it was also suggested by Merat [27] and later by Ajang *et al.* [1] that more protein remains for muscles when fewer feathers are produced, whereas muscle protein and fat contents were not affected by the *Na* gene [28]. According to Cahaner *et al.* [29], a reduction of 1 g feather mass may increase body weight by 1.5 g because feathers contain less water than muscles. There are two other important physiological functions Met may have apart from the protein building block [30]. Met may act as a methyl group donor in methylation processes, but can also be transformed by irreversible degradation into Cys. The latter metabolic pathway is of special significance in diets containing an imbalanced ratio Met:Cys. This question needs more attention in requirement studies for Met, to ensure that degradation of Met to provide Cys is minimized by sufficient dietary Cys supply. In addition, the efficiency of absorption of Cys in the gut and modification of Cys during feed treatments needs generally more attention also in Met requirement studies.

## 4. Conclusions

The estimated optimal Met concentration (percentage of diet) was in the data range given in literature, but influenced by age, aimed PD level, observed Met efficiency (bc^−1^) and feed intake pattern. A further conclusion was that lower feather production did not reduce the requirement for Met in naked neck birds of the genotype under study. However, the reported results of modeling Met requirement data are valid only for the given dietary ratio Met:Cys (1:1) in the experimental diets. Final recommendations for Met requirement data of naked neck birds with special reference to ideal dietary amino acid pattern for these particular genotypes need further investigations. In this context, the role of dietary Cys supply to maximize dietary Met efficiency will gain special attention.

## References

[1] Ajang O., Prijono S., Smith W. (1993). Effect of dietary protein content on growth and body composition of fast and slow feathering broiler chickens. Br. Poult. Sci..

[2] Yalçin S., Özkan S., Açikgöz Z., Özkan K. (1996). Effect of dietary protein content on live and carcase performance of heterozygous naked neck and normally feathered broilers. Br. Poult. Sci..

[3] Nir I. Relationship between feathing and performance in broilers and layers. Proceedings of the 9th European Poultry conference.

[4] Pesti G., Leclercq B., Chagneau A., Cochard T. (1996). Effects of the Naked Neck (Na) gene on the sulfur-containing amino acid requirements of broilers. Poult. Sci..

[5] Samadi F., Liebert F. (2006). Estimation of nitrogen maintenance requirements and potential for nitrogen deposition in fast-growing chickens depending on age and sex. Poult. Sci..

[6] Samadi, Liebert F. (2007). Threonine requirement of slow-growing male chickens depends on age and dietary efficiency of threonine utilization. Poult. Sci..

[7] Samadi, Liebert F. (2008). Modelling the optimal lysine to threonine ratio in growing chickens depending on age and efficiency of dietary amino acid utilisation. Br. Poult. Sci..

[8] Wecke C., Liebert F. (2013). Improving the Reliability of Optimal In-Feed Amino Acid Ratios Based on Individual Amino Acid Efficiency Data from N Balance Studies in Growing Chicken. Animals.

[9] Pastor A., Wecke C., Liebert F. (2013). Assessing the age-dependent optimal dietary branched-chain amino acid ratio in growing chicken by application of a nonlinear modeling procedure. Poult. Sci..

[10] Gous R.M., Morris T.R. (1985). Evaluation of diet dilution technique for measuring the response of broiler chickens to increaseing concentrations of lysine. Br. Poult. Sci.

[11] Khan D.R., Wecke C., Sharifi A.R., Liebert F. (2015). Evaluating the age dependent potential for protein deposition in naked neck meat type chicken. Animals.

[12] Naumann C., Bassler R. (1997). VDLUFA-Methodenbuch. Vol. III. Die chemischen Untersuchungen von Futtermitteln.

[13] German Recommendation of Requirement Standards (Ausschuss für Bedarfsnormen der Gesellschaft für Ernährungsphysiologie) (GRRS) (1999). Empfehlungen zur Energie-und-Nährstoffversorgung der Legenhennen und Masthühner (Broiler).

[14] NRC (1994). Nutrient Requirements of Poultry.

[15] Liebert F., Farke J., Wecke C. Modelling methionine requirements in growing chicken by using the dietary methionine efficiency. Proceedings of 3rd EAAP International Symposium on Energy and Protein Metabolism and Nutrition.

[16] Dirain O., Waldroup P. (2002). Evaluation of lysine, methionine and threonine needs of broilers three to six week of age under moderate temperature stress. Int. J. Poult. Sci..

[17] Kalinowski A., Moran E.T., Wyatt C.L. (2003). Methionine and cystine requirements of slow- and fast-feathering broiler males from three to six weeks of age. Poult. Sci..

[18] Siegel P.B., Dunnigton E.A., Jones D.E., Ubosi C.O., Gross W.B., Cherry J.A. (1984). Phenotypic Profiles of Broiler Stocks Fed Two Levels of Methionine and Lysine. Poult. Sci..

[19] Lehmann D., Pack M., Jeroch H. (1997). Effects of dietary threonine in starting, growing, and finishing turkey toms. Poult. Sci..

[20] Smith E., Pesti G., Bakalli R., Ware G., Menten J. (1998). Further studies on the influence of genotype and dietary protein on the performance of broilers. Poult. Sci..

[21] Rosa A., Pesti G., Edwards H.J., Bakalli R. (2001). Tryptophan requirements of different broiler genotypes. Poult. Sci..

[22] Kidd M., Corzo A., Hoehler D., Kerr B., Barber S., Branton S. (2004). Threonine needs of broiler chickens with different growth rates. Poult. Sci..

[23] Yalcin S., Ozkan S., Acikgoz Z., Ozkan K. (1999). Effect of dietary methionine on performance, carcase characteristics and breast meat composition of heterozygous naked neck (Na/na+) birds under spring and summer conditions. Br. Poult. Sci..

[24] Pesti G., Leclerco B., Cochard T. (1994). Lack of effect on the naked neck gene on the protein requirements of broilers. Poult. Sci.

[25] Deeb N., Cahaner A. (2001). Genotype-by-environment interaction with broiler genotypes differing in growth rate. 1. The effects of high ambient temperature and naked-neck genotype on lines differing in genetic background. Poult. Sci..

[26] Huyghebaert G., Pack M. (1996). Effects of dietary protein content, addition of nonessential amino acids and dietary methionine to cysteine balance on responses to dietary sulphur-containing amino acids in broilers. Br. Poult. Sci..

[27] Merat P. (1986). Potential usefulness of the Na (Naked Neck) gene in poultry production. Worlds Poult. Sci. J..

[28] Cahaner A., Deeb N., Gutman M. (1993). Effects of the Plumage-Reducing Naked Neck (Na) Gene on the Performance of Fast-Growing Broilers at Normal and High Ambient Temperatures. Poult. Sci..

[29] Cahaner A., Dunnington E.A., Jones D.E., Cherry J.A., Siegel P.B. (1987). Evaluation of Two Commercial Broiler Male Lines Differing in Efficiency of Feed Utilization. Poult. Sci..

[30] Pillai P., Fanatico A., Beers K., Blair M., Emmert J. (2006). Homocysteine remethylation in young broilers fed varying levels of methionine, choline, and betaine. Poult. Sci..

